# Discriminating the native structure from decoys using scoring functions based on the residue packing in globular proteins

**DOI:** 10.1186/1472-6807-9-76

**Published:** 2009-12-28

**Authors:** Ranjit Prasad Bahadur, Pinak Chakrabarti

**Affiliations:** 1 Department of Biochemistry, Bose Institute, P-1/12 CIT Scheme VIIM, Calcutta 700 054, India; 2 Current address: Department of Biotechnology, Indian Institute of Technology, Kharagpur 721302, West Bengal, India

## Abstract

**Background:**

Setting the rules for the identification of a stable conformation of a protein is of utmost importance for the efficient generation of structures in computer simulation. For structure prediction, a considerable number of possible models are generated from which the best model has to be selected.

**Results:**

Two scoring functions, R_s _and R_p_, based on the consideration of packing of residues, which indicate if the conformation of an amino acid sequence is native-like, are presented. These are defined using the solvent accessible surface area (ASA) and the partner number (PN) (other residues that are within 4.5 Å) of a particular residue. The two functions evaluate the deviation from the average packing properties (ASA or PN) of all residues in a polypeptide chain corresponding to a model of its three-dimensional structure. While simple in concept and computationally less intensive, both the functions are at least as efficient as any other energy functions in discriminating the native structure from decoys in a large number of standard decoy sets, as well as on models submitted for the targets of CASP7. R_s _appears to be slightly more effective than R_p_, as determined by the number of times the native structure possesses the minimum value for the function and its separation from the average value for the decoys.

**Conclusion:**

Two parameters, R_s _and R_p_, are discussed that can very efficiently recognize the native fold for a sequence from an ensemble of decoy structures. Unlike many other algorithms that rely on the use of composite scoring function, these are based on a single parameter, viz., the accessible surface area (or the number of residues in contact), but still able to capture the essential attribute of the native fold.

## Background

Predicting the native structure of proteins from their amino acid sequences has yet remained an elusive goal. In general this entails the development of effective methods for conformation sampling and the design of an accurate function for structure discrimination [1,2]. The functions could be based on elaborate calculations and analyses of forces between atoms [3,4], or be knowledge-based that extract relevant parameters from a database of experimentally determined protein structures [5,6]. One important area of application of knowledge-based potential functions has been in "protein threading" for the prediction of protein tertiary structure in the absence of detectable sequence homology. The technique involves threading a protein sequence onto the frameworks of known protein folds and finding the most energetically favorable conformation [7-10]. In addition to fold recognition applications, where the best conformation of a protein is selected from a database of known protein conformations, the knowledge-based scoring functions are also used in protein folding simulations [6,11-16]. Many statistical scoring functions assume that frequencies of non-bonded pairs of amino acids follow a Boltzmann-like distribution and the minimum value of the score occurs in the vicinity of the lowest energy structure. Additionally, a set of probability distributions can also be used to construct a scoring function such that it can identify the maximum probability structure.

For testing of empirical energy functions challenging and diverse datasets of decoy structures that are native-like in properties have been generated [12,17-19]. Models submitted in the community-wide experiment, CASP (Critical Assessment of techniques for protein Structure Prediction) [20] make up diverse sets of structures resulting from various computational approaches [21]. The most native-like structure needs to be identified from among these models [22]. An effective potential should be able to distinguish the native structure from decoy structures with a high degree of accuracy. Energy functions based on residue contact or compactness alone do not have enough discriminating power [12], or can rank the native structure highly only when the competing conformations are more random-coil like [23]. However, here we present two knowledge-based scoring functions based on the analysis of residue packing in protein structures that are quite robust in discriminating the native conformation from a number of misfolded conformations for a given primary protein sequence. The functions were also tested on ~ 19000 models from server predictions for 71 targets of CASP7 [20]. As a descriptor for the residue packing we use the average values of the accessible surface area or the number of other residues in contact around a given residue, calculated from a database of globular proteins. Each of the function then evaluates the cumulative value for the deviation of the parameter for individual residues from the corresponding average value over the whole polypeptide chain. The experimental structure is found to have the minimum deviation and thus the minimum value of the function, when applied to a set of decoys from which the native structure has to be identified. The success of the function indicates that the burial of each residue and its contact to the surrounding residues is optimized during folding and the average values of these parameters can be used as constraint to simulate folding process. Additionally, a surface patch with residues having a large overall deviation of these parameters from the average values may be indicative of the binding region on a protein structure, an issue that would be addressed in future to provide a common perception to both the folding and the binding processes.

## Results

Scoring functions have been used to validate X-ray crystal structures, assess and rank three-dimensional models generated for a protein sequence, predict the effect of mutations, etc. Here, we are concerned with the identification of the native structure from decoys. The idea of the use of the discriminatory function originated from the formula of R-factor in crystallography [24]. An exact equivalent formula would have meant the use of the expression (1) instead of (3), given in Methods.(1)

The individual term in Eq. (3) involves the absolute difference between the observed and the average values of ASA for a given residue, normalized by the average value. These terms are summed over the whole sequence. In Eq. (1) the numerator and the denominator are summed separately. Some other modified formulae, including the use of the standard deviation on the average values <ASA_x_> in the denominator, were also tried, but (2) and (3) were found most efficient to identify the native structure from a set of decoys. Depending on the structural context larger residues may have a considerable variation in their ASA values in protein structures (as indicated by larger standard deviations, Table 1) - normalization of the difference in the numerator in Eq. (3) has the effect of damping the contribution of such residues in the summation.

**Table 1:** Average values of partner number (<PN>) and accessible surface area (<ASA>) of different amino acid residues

Residue	<PN>	<ASA>
Gly	7.4 (2.2)	26.6 (24.5)
Ala	8.6 (2.5)	28.1 (30.9)
Ser	7.9 (2.6)	39.2 (33.2)
Cys	10.0 (2.3)	17.1 (21.0)
Thr	8.5 (2.6)	44.2 (36.0)
Asp	7.9 (2.5)	58.1 (37.2)
Pro	7.7 (2.6)	54.2 (39.5)
Asn	8.3 (2.7)	57.9 (40.8)
Val	10.3 (2.6)	24.1 (32.0)
Glu	8.4 (2.5)	73.4 (41.9)
Gln	9.0 (2.7)	68.6 (43.3)
His	9.7 (2.9)	53.8 (44.6)
Leu	11.0 (2.7)	28.8 (38.0)
Ile	11.0 (2.7)	25.0 (35.2)
Met	11.2 (3.1)	35.5 (45.8)
Lys	8.4 (2.4)	95.8 (42.9)
Phe	11.9 (2.9)	31.0 (39.8)
Tyr	11.5 (3.1)	45.5 (45.0)
Arg	10.1 (3.1)	85.5 (53.3)
Trp	12.6 (3.2)	43.5 (47.6)

Data taken from [26]. The standard deviations are in parenthesis.

### Quantification of the overall packing of residues in protein structures

The average number of partner residues and the average accessible surface area for all twenty amino acids are provided in Table 1[25]. While the <ASA> values are almost identical to those calculated earlier [26], the values for the partner number are different, as the calculation is residue-based here, while in the earlier study the individual atoms constituted the partners.

As R_p _and R_s _indicate the extent of deviation of PN and ASA of residues from their average values, taken over the whole structure, these parameters can be used to judge the optimization of packing of residues in a structure [27]. We also wanted to see if there is any variation depending on the class of protein. However, as R_p _and R_s _provide cumulative values over all the residues in a structure, it is sensible to divide them by the number of residues in a structure before comparison. Individual protein structures in the dataset were classified according to CATH (Class, Architecture, Topology, Homologous superfamily; http://www.cathdb.info/index.html) into 157 all-α, 142 all-β and 133 αβ (including α+β and α/β) classes of proteins. The normalized values (Table 2) are rather similar, except slightly higher values in the all-β class, indicating somewhat higher deviations from the optimum values of PN and ASA in these structures. The observation of higher values in β-proteins is in tune with a relatively lesser packing efficiency in these proteins, as is also demonstrated by the higher occurrence of cavities involving residues in β-sheets [28].

**Table 2:** Average values of R_s _and R_p _in various protein structural classes^a^

	Number of structures	R_s_	R_p_
All-α	157	112 (63)	30 (20)
		*0.92 (0.86)*	*0.27 (0.27)*
			
All-β	142	115 (60)	31 (19)
		*1.26 (1.01)*	*0.37 (0.33)*
			
αβ ^b^	133	149 (70)	42 (23)
		*0.72 (0.55)*	*0.23 (0.18)*
			
Overall	432	143 (91)	39 (23)
		*0.83 (0.76)*	*0.22 (0.17)*

^a^According to CATH [59]. ^b^Including a/β and a+β.

Standard deviations are in parentheses. Normalized (dividing the values obtained from equations (2) and (3) by the number of residues) values are given in italics.

### Identification of the native structure from misfolded decoys

#### PROSTAR decoy sets

The objective of this work is to discriminate between the native structure and one or more misfolded or low-resolution structures. The utility of R_p _and R_s _was tested on the decoy sets in the PROSTAR website and the results are shown in Table 3. When compared with other atomic or residue-based potentials, the present parameters, R_s _and R_p _have similar or better performance, except for 'Ifu'. Of the two parameters, R_s _based on residue accessibility performs better than the one derived on the basis of partner number (R_p_).

**Table 3:** Identification of the native structure from decoys in PROSTAR decoy sets using different scoring functions^a^

Parameters	Misfold	Ifu	Asilomar	Pdberr and sgpa
R_s _^b^	24/24	22/43	41/41	5/5
R_p _^b^	20/24	21/43	36/41	5/5
Atomic KBP^c^	24/24	32/43	37/41	5/5
RAPDF^d^	24/24	30/43	37/41	5/5
CDF^d^	19/24	21/43	35/41	5/5
Residue contact potential^e^	24/24	22/43	35/41	4/5

PROSTAR website [31].

^a^The first number of each column is the number of correctly identified decoys, and the second one after the slash is the total number of decoys. With either of the first two parameters the native structure is correctly identified if its value is smaller than that from any other structure in the decoy set. The results with the other parameters are taken from [14].

^b^The parameters developed in this study.

^c^The atomic Knowledge-Based Potential from Lu and Skolnick [14].

^d^RAPDF and CDF are atomic and residue-based potentials, respectively, from Samudrala and Moult [13].

^e^Residue-based quasichemical potential from Skolnick et al[33].

The 'Misfold' decoy set, generated by Holm and Sander [17], consists of 24 examples of pairs of proteins with the same number of residues in the chain, but different sequences and conformations. Sequences are swapped between members of a pair, resulting in rather inappropriate environments for most of the side chains. For this set, R_s _selects 100% of the structures correctly, but R_p _fails in four. Attempts were made to see if the use of other cut-off distances (4.0, 5.0, 6.0 and 7.0 Å) in the definition of R_p _improved the situation, but the performance of the parameter derived at 4.5 Å was found to be the best.

The 'Ifu' decoy set is based on a set of 43 peptides, 10-20 residues long, which are proposed to be independent folding units as determined by local hydrophobic burial and experimental evidence [29]. In this test set, R_s _and R_p _were unsuccessful to pick 21 and 22, respectively, out of 43 native structures. While performing the best, even the knowledge-based potential [14] failed in 11 cases in this test set. This is probably because the targets in these subsets are protein pieces and it is difficult for residue packing parameters derived from larger proteins to evaluate these structures.

The 'Asilomar' decoy set resulted from the first experiment on the Critical Assessment of Protein Structure Prediction methods (CASP), which produced a set of 41 comparative models of six different proteins [30]. The models vary in C^α ^rmsd to the corresponding experimental conformation, ranging from 0.53 to 7.40 Å, depending on the difficulty of the model building process. In this test set, the parameter R_s _selects 100% native structures correctly, by far the best result from any discriminatory function. For R_p_, missing 5 out of 41 cases, the performance is at par with other functions.

The 'Pdberr' decoy set consists of structures determined using X-ray crystallography that were later found to contain errors, and the corresponding corrected experimental conformations [31]. The 'sgpa' decoy set consists of the experimental structure *Streptomyces griseus *Protease A (2sga) and two conformations generated by molecular dynamics simulations starting with the experimental structure [32]. In these test sets, where the decoys are low-resolution X-ray structures, both the scoring functions R_s _and R_p _correctly picked the high-resolution structures in all cases, as did all other potential functions, except the one based on the residue contact potential with a composition-corrected scale [33].

#### Park and Levitt decoy set

**Figure 1:**
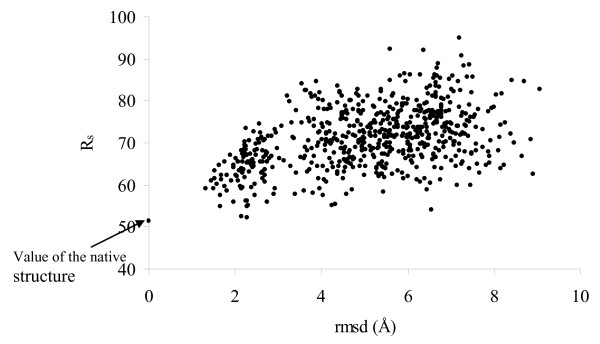
**Scatter plot of R_s _vs. rmsd for a representative protein structure, **1ctf, **along with its decoys**.

The Park and Levitt decoy test set, available on the web site http://dd.compbio.washington.edu, consists of 7 sequences, each with nearly 600-700 decoys that cover structures showing an rmsd ranging from 0 (the correct fold) to 10 Å from the native structure [12]. The protein structures were generated by using four-state models (four discrete ϕ,ψ angles) to define the conformation of each of ten selected residues in each protein using an off-lattice model. From the very large number of conformations generated, only those compact structures were retained that scored well using a variety of scoring functions, as well as having a reasonable rmsd from the native structure. The 4-state-reduced decoy data set given in Additional file 1: Table S1 includes a range of small proteins from 54-75 residues with varying topological folds, with the numbers of decoys ranging from 630 for 1ctf to 687 for 4pti. A positive Z-score (Equations (4) and (5)) indicates that the value of the parameter for a particular native fold is lower than the average of the distribution. While considering the Z_s_, the native structure is well separated from the average of the distribution for all the structures, but Z_p _shows an inferior result for 1r69 and 1sn3. Figure 1 plots R_s _vs rmsd for a representative dataset corresponding to the PDB file, 1ctf. The value of R_s _is the minimum for the native structure. There is a good linear correlation between the two variables (R^2 ^is 0.78), better than that (0.6) obtained using the knowledge-based potential of Lu and Skolnick [14]. While the various energy functions based on empirical contact, surface area and van der Waals energy did not perform consistently well to distinguish between correct and incorrect conformations and had to be used in combination for the proper identification of the correct fold [12], the rather simple parameter, R_s _has a remarkable discriminatory power.

The Levitt low-minima decoy sets (LMDS) also contain structural decoys (the number ranging from 343 to 500) for 7 small proteins, 36 to 68 residues long [19]. From an initial ten thousand structures, generated by randomly modifying only the loop dihedral angles, which were subjected to minimization using a modified ENCAD force field involving united and soft atoms [34], up to five hundred of the lowest energy conformations were retained to make up the decoy sets. For all the 7 cases the native structure has the minimum R_s _value and the corresponding Z-score indicates that it is well separated from the decoys (Additional file 1: Table S1). However, Z_p _gives an inferior result for 1bba and 1fc2. Other energy functions also failed to identify the native structure for these two proteins [15,22] due to the fact that the native conformation is simply not very well defined for the former [35] and the latter is a fragment of a larger protein and additionally, a constituent of a complex, and in the unbound form may have a structure different from that in the complex [36]. Interestingly however, based on R_s _both the native structures are separated by about two standard deviations from the average of the distribution.

#### ROSETTA decoy sets

The ROSETTA all-atom decoy sets are composed of five different proteins ranging in size from 92 to 116 residues, and the number of decoys ranging from 994 to 999 (Additional file 1: Table S1) [37]. Fragments, between 3 and 9 residues, from known structures matched to the targets through a multiple sequence alignment process, were assembled into the protein structures via the fragment insertion-simulated annealing strategy [37]. The scoring functions used to select the lowest energy decoys included hydrophobic burial, electrostatics, the formation of β-sheets and the packing of α-helices and β-strands. The Z-scores based on R_s _and R_p _indicate that both the scoring functions perform well over all the 5 structures. The large Z-scores seen here, as compared to those in others, should be due to the high rmsds in the decoys used in this test set.

The original ROSETTA decoy set has been improved by increasing the number of proteins and frequency of near native models, providing 1,400 model structures for 78 diverse, single domain proteins with varying degrees of secondary structure and length from 25 to 87 residues for the evaluation of scoring functions [16]. The discriminatory ability of our scoring functions can be seen from the results on 41 cases (a subset of the complete dataset, which is downloadable) presented in Additional file 1: Table S2. The native structure did not have the minimum R_s _value in 3 cases, while R_p _failed in two additional cases. For these, the Z-score is also quite small, Z_p _even registering a negative value in two. It may be noted that two structures (1res and 1uxd) among the failed cases were derived from NMR experiments and the Rosetta energy functions are also less efficient in identifying the NMR structures as compared to X-ray crystal structures, probably because the former structures have greater deviation of side chain conformations from the canonical rotamer conformations [16].

#### Identification of the native structure from the native-like conformation constructed by homology modeling

Samudrala and Levitt [19] have a decoy set (hg_structal) for 29 globins. Each globin has been built by comparative modeling using 29 other globins as templates with the program segmod [38]; the rmsd for the modeled structures range from 1.96 to 8.57 Å. A similar decoy set (ig_structal_hires) involving 20 immunoglobulins and at a relatively higher resolution (1.7-2.2 Å, compared to the range of 1.7-3.1 Å for the full set of 61 proteins) is also available. The application of our scoring function on these two sets yields results given in Table 4. As with the other decoy sets, R_s _performs better than R_p _in identifying the native structure. Even though the homology built models in the 'ig_structal_hires' set are very close to the native structure, the latter was identifiable in 90% of the cases.

**Table 4:** Identification of native structure from decoys constructed by homology modeling

Parameters	hg_structal	ig_structal_hires
R_s_	23/29	18/20
R_p_	15/29	17/20

Dataset taken from [19]http://dd.compbio.washington.edu. The first column in each category is the number of correctly identified decoys, and the second column is the total number of decoys.

#### Score of the experimental structure relative to the solutions submitted to CASP7

The ability of our scoring function to identify the native structure from the best near-native solutions has been tested on the CASP7 dataset [20]. This is the most difficult test as the decoys are the best predicted near-native structures submitted by different groups participating in the CASP experiment. CASP7 experiment consists of 95 accepted targets for which about 22000 models were submitted. We have excluded the NMR structures and retained 71 targets (with ~ 19000 models) to evaluate our scoring functions (Additional file 1: Table S3). The rmsd between the native structure and the best predicted solution varies in the range 0.4 - 2.6 Å in the whole dataset. Z_s _identifies the best solution in 51 cases and Z_p _in 38. Table 5 compares the results of our study vis-à-vis those from other algorithms [22]. As we have seen before, R_s _performs better than R_p_. But even R_p _outperforms other existing functions in locating the native structure among the top ten solutions. R_s _identifies the native structure as the top solution in 72% of cases, which is considerably better than the next best performer (DFIRE and QMEAN3) at 62%.

**Table 5:** Performance of the different scoring function for predicting the native structure among the best near-native structures submitted in CASP7

		% of the native structure^b^	
		
Method^a^	Z_nat_	Rank1	Rank10
Modcheck	1.99	49.47	72.63
RAPDF	-2.09	57.89	81.05
DFIRE	-1.25	62.11	75.79
ProQ	1.51	9.47	33.68
ProQ_SSE	1.76	14.74	44.21
FRST	-2.41	58.95	75.79
QMEAN3	-2.27	62.11	78.95
R_p_	1.69	53.52	91.55
R_s_	2.17	71.83	98.59

Z_nat _corresponds to the average Z-score of the native structure.

^a ^Except the last two functions, the performance of others are based on the data provided in Table 6 of [22].

^b ^% of the native structure with rank 1 or within rank 10 from among all the solutions submitted in CASP7.

## Discussion

There are many energy functions (knowledge based statistical scoring function or physics-based or a combination of both) which find the correct native conformation from misfolded decoys [3,6,9,12-15,22,39-42]. However, it is rather nontrivial to develop a function that works across different decoy sets and a combination of functions is normally used [12,13]. R-factor is the gold-standard for expressing the accuracy of crystallographic analysis, and as knowledge-based functions are mostly "trained" on crystal structures it is rather gratifying to develop functions similar to R-factor that can also be used to characterize the native structure (Table 2).

The present study demonstrates the development of scoring functions from the properties of residue packing that can be useful for discriminating the native conformation from various misfolded conformations for a given protein sequence. The algorithm assumes that a protein tries to take up a fold that has the minimum deviation of ASA (or PN) of each residue from the average value observed over all protein structures. The function R_s_, based on residue accessibility, performs better than the one derived from the partner number, R_p_, on decoy sets. The test on various decoy sets from the PROSTAR website demonstrated that the knowledge based scoring function developed in this study performs better or even at least of the same order than those previously derived by many authors [12,14,15]. Not only the present knowledge-based scoring functions pick the correct native structure in most cases, but the discrimination ratio is also better than that of the other potentials. However, as Equations (2) and (3) use the average values derived from a database of globular proteins, it is not likely to be very discriminatory for small proteins or peptides (as seen for the 'Ifu' set in Table 3). As such it would not be useful for checking local model quality in protein structures, as done by packages such as PROSA [43]. Along the same line it may be mentioned that the Verify3D server [44] for the visual analysis of the quality of a crystal structure works best on proteins with at least 100 residues.

The Park and Levitt decoy set had been shown to be quite a challenging dataset where the lowest-energy structures typically were 6-10 Å rmsd away from native ones [12]. The improved residue-based potential [18] also cannot recognize the native and near-native structures in all cases. The knowledge based scoring functions derived in this study are quite efficient to identify the near-native fold in Park and Levitt decoy sets. The correlation between the scoring function and rmsd is good in all cases and most of the cases the scoring functions have minimum value for the native structure. The scoring functions perform well also in the PROSTAR decoy sets, Levit's Local-Minima Decoy Sets (LMDS) and also in ROSETTA All-atom Decoy Sets. Considering 222 independent cases considered in this analysis R_s _and R_p _can efficiently discriminate native structures from all their corresponding decoys with a success rate greater than 85% and 74%, respectively. If we do not consider the 'Ifu' dataset, which comprises of small fragments of polypeptide chains, the success rate increases to 94% and 80%, respectively. The most rigorous test of a scoring function is to evaluate its performance in identifying the native structure with reference to the models submitted in CASP7 experiment. Even here, both R_s _and R_p_, the former in particular, stand out from all other methods (Table 5).

As our scoring functions depend on ASA or PN, these should be closely related to potentials of mean force derived from solvation or packing considerations. The performance of these potentials, however, depend critically on how the standard state is specified [6,12,23]. As the core and surface regions in proteins constitute distinct environments, potentials are sometimes divided into two parts, for the buried and the solvent-accessible regions [40]. The use of the average values of ASA or PN in globular proteins seems to have eliminated the need of such division, or the debate on the proper choice of the standard state.

A discussion on the uniqueness of our parameters vis-à-vis other knowledge-based discrimination functions is in order. First, a residue in the sequence is normally represented in these functions with one or two positions in the three-dimensional space and one or more of its properties, such as the secondary structure or backbone dihedral angle preferences, features in distance or sequence separation from other residues, etc. are considered [7,23]. With such a coarse representation the function may not be as efficient as an all-atom discriminatory function, which takes into account the environment of all the atoms in a residue [13,45-47]. An all-atom representation is implicit in our method, as all the atoms are needed for the calculation of ASA or the partner number. However, each residue in the sequence contributes singly to the derivation of R_s _or R_p_. This is also in contrast to residue-residue interaction energy for each residue pair that is normally employed in other functions [12,48,49]. Furthermore, residue triplets and four-body contact potentials have also been developed [50,51]. Secondly, the energy functions are generally less discriminatory when used individually, and the use of the hybrid scoring function is the norm for an enhanced performance [12,16,22]. While conceptually simple, R_s _or R_p _can work as efficiently. Thirdly, most formulations use energy as the criterion (with the assumption that the native structure is at a global free-energy minimum), while our function seeks to find the conformation that has the minimum deviation from the average value of the partner number or ASA. This way the selection of the most compact state of the polypeptide chain corresponding to a given sequence is achieved. The parameters are less likely to be fooled by over-abundance (which is penalized to the same extent as lower-abundance in equations 2 and 3) of contacts, as is the case with some functions [12]. Lastly, as the functions can identify the correct structure from the erroneous ones modeled from X-ray data ('Pdberr' set in Table 3) and vary within a narrow range in different protein classes (Table 2), these can be used for the validation of the structure determined crystallographically [52].

The functions developed here can also be used to delineate the compatibility of the sequence to a fold For example, azurin [53] and plastocyanin [54] are two small proteins having the same fold (a sandwich of two β-sheets having seven strands), but sequence identity of only 17% over an aligned length of 86 residues (Table 6). Expectedly, they have very similar R_s _and R_p _values. More interestingly however, when the sequence of plastocyanin is considered over the structure of azurin one gets a value of 0.97 for (R_s_)_azu/pcy_, quite close to 0.89 obtained for the reverse process ((R_s_)_pcy/azu_), thereby indicating the compatibility of the two sequences to the same fold.

**Table 6:** R_s _and R_p _for two proteins having the same fold belonging to the β class

Name of the protein	Number of residues	Number of aligned residues	R_s_	R_p_
Azurin (1azu)	126	84	1.12 *(1.06)*	0.33 *(0.29)*
Plastocyanin (5pcy)	99	84	1.33 *(1.14)*	0.46 *(0.32)*

The structures are aligned using the software SSM at EBI http://www.ebi.ac.uk/msd-srv/ssm. The values calculated considering only the aligned amino acid residues are given in parenthesis. To quantify the sequence structure compatibility between the structures, two more parameters are computed over the aligned residues. (R_s_)_azu/pcy _= 0.97 and (R_s_)_pcy/azu _= 0.89. Each term contributing to the former corresponds to (ASA_azu _- <ASA>_pcy_)/<ASA>_pcy_, i.e., in Eq. (3) the observed value at a given position in the structure of azurin is compared to the average value corresponding to the aligned residue type at the same position in the sequence of plastocyanin. The opposite is done in the calculation of (R_s_)_pcy/azu_.

## Conclusion

This work demonstrates the effectiveness of a simple knowledge-based scoring function derived from residue packing for discriminating the native structures from a large set of decoys constructed by several groups. This knowledge-based scoring scheme is simple to derive and less computationally intensive than other energy functions and the performance is better (or at least at par) compared to others. Used in conjunction with other chemically intuitive parameter that captures the essence of the protein structure, it should be possible to achieve complete discrimination between the native structure and decoys.

## Methods

Atomic coordinates were obtained from the Protein Data Bank (PDB) [55]. The analysis was carried out using the dataset of 432 polypeptide chains in 418 PDB files (given in [26]) with an *R*-factor ≤ 20%, a resolution ≤ 2.0 Å and sequence identity < 25%. Also the polypeptide chains with >40% of atoms with temperature factor (*B*-factor) >30 Å^2 ^were excluded. The calculation of the partner number was restricted only to the well-ordered residues by excluding those with >40% atoms with temperature factor >30 Å^2^. The solvent accessible surface area (ASA) was computed using the program NACCESS [56], which is an implementation of the Lee and Richards algorithm [57]. The partner number of a residue is the number of other residues within a distance of 4.5 Å from any atom of the residue under consideration; the flanking residues were not considered as partner if the interaction was only with the main-chain atoms. The reason for the selection of the particular threshold value for the distance has been discussed [26,58]. To be identified as a partner it is enough if just a pair of atoms is in contact.

Two parameters R_p _and R_s _based on the observed partner number and the accessibility at a given position in the protein sequence, as compared to the average value of the parameters for the same residue type in the whole database, were developed as given in the following two equations(2)

where PN_xi _and ASA_xi _are the observed partner number and the solvent accessible surface area, respectively, for a residues of type x occurring at a particular position, i, in a PDB file and <PN_x_> and <ASA_x_> are the average values of the residue type x in the analyzed dataset. Considering (3), the function sums up the absolute value of the deviation of ASA at each position in the sequence from the average ASA of the residue type, each term being normalized by the average ASA value. The magnitude of each of the two parameters derived using (2) and (3) is used to discriminate the near native fold from the misfolded decoys. For the correct fold the values of these two parameters should be minimum.

A number of decoy datasets have been used from literature, the details of which are provided in Results. The Z-score of a native structure and the misfolded decoys was also evaluated. The Z-scores using the residue accessibility (Z_s_) and residue partner number (Z_p_) of a particular protein conformation are defined by the following equations(4)

where R_s-nat _(or R_p-nat_) is the value of the parameter for the native conformation, and <R_s_> (<R_p_>) and σ are the average and the standard deviation of the distribution of the parameter in the set. The magnitude of the Z-score is an indication of how far that native conformation is separated from the near native structures in the distribution.

## Authors' contributions

PC conceptualized the work that was carried out by RPB. RPB and PC participated in interpretation of the data and writing the manuscript. Both the authors read and approved the final manuscript.

## Supplementary Material

Additional file 1 **Identification of native structure from decoys in different decoy sets**. The file contains three tables, numbered S1 to S3. Click here for file
