# Supplementary material for: Discriminating the native structure from decoys using scoring functions based on the residue packing in globular proteins
Source: BMC Struct Biol. 2009 Dec 28;9:76. doi: 10.1186/1472-6807-9-76 (PMC2809062; doi:10.1186/1472-6807-9-76)
Supplement: Additional file 1 — Identification of native structure from decoys in different decoy sets. The file contains three tables, numbered S1 to S3. [file 1472-6807-9-76-S1.DOC]

**Table S1.** Identification of native structure from decoys

| Decoy set | PDB | Description | N_res_^a^ | N_dec_^b^ | rmsd range | Z_s_/rank^c^ | Z_p_/rank^c^ |
| --- | --- | --- | --- | --- | --- | --- | --- |
| 4-state-reduced | 1ctf | C-terminal domain of ribosomal protein L7/L12 | 68 | 630 | 2.16-10.16 | 2.7 | 2.0 |
|  | 1r69 | N- terminal domain of phage 434 repressor | 63 | 675 | 2.28-9.50 | 2.2 | 0.6/19 |
|  | 1sn3 | Scorpion toxin variant 3 | 65 | 660 | 2.50-10.46 | 1.4/5 | 0.7/23 |
|  | 2cro | Phage 434 Cro protein | 65 | 674 | 2.05-9.72 | 4.6 | 3.7 |
|  | 3icb | Vitamin D-dependant calcium-binding protein | 75 | 653 | 1.81-10.74 | 1.7 | 1.2/6 |
|  | 4pti | Trypsin inhibitor | 58 | 687 | 2.83-10.79 | 3.8 | 2.7 |
|  | 4rxn | Rubredoxin | 54 | 677 | 2.58-9.28 | 3.1 | 3.5 |
|  |  |  |  |  |  |  |  |
| LMDS^d^ | 1bba | Pancreatic hormone (ave. NMR) | 36 | 500 | 3.98-9.98 | 2.1 | 0.6/19 |
|  | 1ctf | C-terminal domain of ribosomal protein L7/L12 | 68 | 497 | 4.40-12.92 | 2.8 | 2.2 |
|  | 1fc2 | Fragment B of protein A (complexed to immunoglobulin Fc) | 43 | 500 | 5.12-9.42 | 1.7 | 0.8/27 |
|  | 1igd | 3rd IgG-binding domain from streptococcal protein G | 61 | 500 | 4.45-13.11 | 4.3 | 3.5 |
|  | 2cro | Phage 434 Cro protein | 65 | 500 | 5.09-14.13 | 3.3 | 2.7 |
|  | 2ovo | 3rd domain of silver pheasant ovomucoid | 56 | 347 | 5.64-14.14 | 5.2 | 3.3 |
|  | 4pti | Trypsin inhibitor | 58 | 343 | 5.93-14.06 | 2.9 | 2.3 |
|  |  |  |  |  |  |  |  |
| ROSETTA | 1ksr | F-actin cross-linking gelation factor | 92 | 998 | 9.82-24.09 | 4.8 | 3.3 |
|  | 1lzl | Lysozyme | 116 | 999 | 9.57-30.11 | 4.2 | 3.6 |
|  | 1ris | Ribosomal protein S6 | 92 | 999 | 7.38-22.43 | 5.3 | 4.6 |
|  | 1tul | Telokin-like protein | 97 | 999 | 10.72-35.12 | 3.3 | 3.1 |
|  | 2acy | Acylphosphatase | 92 | 994 | 8.83-28.23 | 4.4 | 3.9 |

Datasets used are: 4-state-reduced [12], LMDS [19] and ROSETTA [18].

^a^ Number of residues. ^b^ Number of decoys. ^c^ The rank for the native structure is provided, if different from 1, after a slash.

^d^ Four (with codes 1b0n, 1dtk, 1shf and smd3) out of 11 structures in the decoy set have been excluded as they are either NMR structure or their atoms have high temperature factors (see Methods).

**Table S2.** Identification of native structure from decoys in new, all-atom ROSETTA decoy set

| PDB | Description | N_res_ | N_dec_ | rmsd | Z_s_/rank | Z_p_/rank |
| --- | --- | --- | --- | --- | --- | --- |
| 1res | γδResolvase (DNA Binding Domain) | 35 | 1723 | 1.025 | 1.8/3 | 0.6/11 |
| 1uxd | Fructose Repressor DNA-Binding Domain | 43 | 1896 | 1.123 | 2.3 | 0.8/13 |
| 2pdd | Oxido-Reductase(Acyltransferase) | 43 | 1740 | 2.329 | 2.6 | 1.8 |
| 1uba | DNA Repair Protein Hhr23A | 45 | 1899 | 2.997 | 2.8 | 1.7 |
| 1gab | Albumin-Binding Protein | 47 | 1898 | 1.447 | 2.6 | 1.4 |
| 1bw6 | Human Centromere Protein B (Cenp-B) DNA Bindign Domain Rp1 | 56 | 1900 | 1.894 | 2.3 | 1.6 |
| 1am3 | HIV Capsid C-Terminal Domain | 57 | 1898 | 1.356 | 1.8 | 1.3 |
| 1r69 | 434 Repressor (Amino-Terminal Domain) | 61 | 1733 | 1.372 | 1.6 | 1.6 |
| 1utg | Uteroglobin (Oxidized) | 62 | 1897 | 3.364 | 2.8 | 2.3 |
| 1a32 | Ribosomal Protein S15 | 65 | 1610 | 0.917 | 0.6/7 | -1.6/29 |
| 2ezh | I gamma subdomain of the Mu end DNA-binding domain of phage Mu transposase | 65 | 1893 | 2.335 | 2.2 | 1.6 |
| 1nre | Receptor-Associated Protein | 66 | 1893 | 1.802 | 2.3 | 1.4 |
| 1ail | N-Terminal Fragment Of Ns1 Protein From Influenza A Virus | 67 | 1807 | 1.971 | 2.3 | 1.8 |
| 1lfb | Transcription Factor Lfb1 (Homeodomain) | 69 | 1893 | 2.471 | 1.8 | 2.1 |
| 1nkl | Nk-Lysin | 70 | 1898 | 2.732 | 2.9 | 2.4 |
| 1pou | Oct-1 (Pou-Specific Domain) | 70 | 1898 | 2.278 | 2.3 | 2.8 |
| 1mzm | Maize Nonspecific Lipid Transfer Protein Complexed With Palmitate | 71 | 1934 | 2.692 | 2.4 | 2.3 |
| 1kjs | C5A | 74 | 1893 | 3.138 | 2.8 | 3.2 |
| 1hyp | Hydrophobic Protein From Soybean | 75 | 1893 | 4.052 | 3.2 | 2.7 |
| 1cc5 | Cytochrome C_5_ (Oxidized) | 76 | 1892 | 4.311 | 3.4 | 2.8 |
| 1cei | Colicin E7 Immunity Protein | 85 | 1897 | 4.572 | 3.6 | 3.8 |
| 1ptq | Protein Kinase C δ Cys2 Domain | 43 | 1885 | 5.418 | 3.3 | 3.6 |
| 1aa3 | C-Terminal Domain Of The E. Coli Reca | 56 | 1865 | 2.122 | 3.2 | 2.7 |
| 1orc | Cro Repressor Insertion Mutant K56-[Dgevk] | 56 | 1883 | 2.81 | 2.7 | 1.8 |
| 1pgx | Protein G Type 7 (B2 Domain) | 57 | 1851 | 1.481 | 2.2 | 0.8/17 |
| 1tif | Translation Initiation Factor 3 N-Terminal Domain | 59 | 1849 | 2.605 | 2.5 | 1.4 |
| 2ptl | Immunoglobulin Light Chain-Binding Domain of Protein L | 60 | 1835 | 2.208 | 2.6 | 1.8 |
| 1dol | Monocyte Chemoattractant Protein 1, I-Form | 62 | 1871 | 3.766 | 2.8 | 2.9 |
| 2fow | RNA Binding Domain Of Ribosomal Protein L11 | 66 | 1834 | 2.666 | 2.6 | 2.7 |
| 1ctf | L7/L12 50 S Ribosomal Protein (C-Terminal Domain) | 67 | 1922 | 2.657 | 1.8 | 2.2 |
| 1afi | Mercuric Transport Protein | 72 | 1824 | 2.243 | 2.3 | 1.8 |
| 5icb | Vitamin D-Dependent Calcium-Binding Protein | 72 | 1870 | 2.98 | 1.9 | 1.4 |
| 1vcc | DNA Topoisomerase I | 77 | 1857 | 3.845 | 3.2 | 2.6 |
| 2fxb | Oxidized [4Fe-4S] Ferredoxin | 81 | 1800 | 5.484 | 3.8 | 3.6 |
| 1vif | Dihydrofolate Reductase | 48 | 1896 | 0.443 | 0.3/13 | -1.4/23 |
| 1bq9 | Rubredoxin (Formyl Methionine Mutant) | 53 | 1825 | 2.793 | 1.8 | 1.2 |
| 5pti | Trypsin Inhibitor (Crystal Form II) | 55 | 1853 | 3.941 | 2.2 | 2.8 |
| 1msi | Type III Antifreeze Protein Isoform Hplc 12 | 60 | 1894 | 5.409 | 3.4 | 3.3 |
| 1tuc | α-Spectrin | 61 | 1894 | 4.485 | 3.6 | 3.8 |
| 1csp | Major Cold Shock Protein (Cspb) | 64 | 1809 | 3.241 | 3.7 | 2.9 |
| 1sro | S1 RNA Binding Domain | 66 | 1881 | 2.689 | 2.6 | 2.3 |

Dataset taken from [16] (website, http://depts.washington.edu/bakerpg/decoys/). Columns heads are explained in Table S1, except that rmsd here represents the value for the decoy structure closest to the native.

**Table S3**. Identification of the near native structure in the CASP7 dataset

| CASP7 ID | PDB ID | Description | N_res_ | N_prd_^a^ | rmsd | Z_s_/rank | Z_p_/rank |
| --- | --- | --- | --- | --- | --- | --- | --- |
| T283 | 2HH6 | BH3980 protein from *Bacillus Halodurans* | 113 | 541 | 1.5 | 1.87 | 1.52 |
| T284 | 3B8I | Oxaloacetate decarboxylase from *Pseudomonas Aeruginosa* | 287 | 483 | 1.3 | 1.48 | 1.02 |
| T285 | 2VA0 | Extracytoplasmic domain from histidine kinase, *Cellvibrio Japonicus* | 131 | 514 | 2.3 | 2.67 | 2.20 |
| T286 | 2VPT | Carbohydrate esterase from *Clostridium Thermocellum* | 215 | 515 | 1.6 | 2.82 | 2.06 |
| T287 | 2G3V | CAG pathogenicity island protein | 208 | 458 | 1.9 | 1.34 / 2 | 1.20 / 6 |
| T288 | 2GZV | PDZ domain of human PICK1 | 114 | 531 | 1.4 | 0.89 / 4 | 0.59 / 7 |
| T289 | 2GU2 | Aspartoacylase | 312 | 479 | 2.1 | 1.13 / 6 | 0.69 / 19 |
| T290 | 2GW2 | peptidyl-prolyl isomerase domain of human cyclophilin G | 198 | 488 | 0.5 | 1.37 / 3 | 1.22 / 6 |
| T291 | 2GSF | Human Epha3 receptor tyrosine kinase | 373 | 482 | 0.7 | 2.67 | 2.35 |
| T292 | 2JAV | Human Nek2 centrosomal kinase | 279 | 464 | 1.7 | 3.89 | 3.88 |
| T293 | 2H00 | Human methyltransferase | 254 | 476 | 2.0 | 3.98 | 3.61 |
| T296 | 2HA9 | SP0239 from *Streptococcus Pneumoniae* | 446 | 555 | 2.3 | 2.48 | 1.54 / 9 |
| T297 | 2HSJ | Platelet activating factor from *Streptococcus pneumoniae* | 214 | 512 | 1.8 | 1.91 | 1.20 / 6 |
| T299 | 2HIY | SP0830 from *Streptococcus Pneumoniae* | 183 | 499 | 2.6 | 1.10 / 4 | 0.90 / 10 |
| T300 | 2H3R | Murid herpesvirus 4 (MuHV-4) | 110 | 575 | 0.7 | 1.61 | 1.40 |
| T301 | 2H9F | hypothetical protein (np_249484.1) from *Pseudomonas Aeruginosa* | 396 | 491 | 2.0 | 3.13 | 1.78 / 10 |
| T303 | 2HSZ | Predicted phosphatase from *Haemophilus Somnus* | 243 | 482 | 1.8 | 1.82 | 1.20 |
| T304 | 2H28 | YeeU from *E. Coli* | 130 | 543 | 1.9 | 3.89 | 3.38 |
| T305 | 2H4V | Human tyrosine receptor phosphatase gamma | 320 | 528 | 0.9 | 1.89 | 1.38 |
| T306 | 2HD3 | Ethanolamine Utilization Protein EutN from *E. Coli* | 103 | 542 | 2.1 | 2.88 | 2.55 |
| T307 | 2H5N | Hypothetical protein PG_1108 from *Porphyromonas Gingivalis* | 133 | 531 | 2.4 | 0.82 / 7 | 0.68 / 12 |
| T308 | 2H57 | Human ADP-ribosylation factor-like 6 | 190 | 513 | 1.2 | 1.56 | 1.35 |
| T309 | 2H4O | Hypothetical protein yonK from *Bacillus Subtilis* | 76 | 538 | 1.8 | 2.99 | 2.43 |
| T311 | 2ICT | Bacterial antitoxin HigA from *E. Coli* | 94 | 541 | 0.9 | 0.78 / 3 | 0.16 / 6 |
| T312 | 2H6L | Metal-containing protein AF0104 from *Archaeoglobus Fulgidus* | 146 | 502 | 1.7 | 1.13 / 3 | 0.80 / 10 |
| T313 | 2H58 | KIFC3 motor domain in complex with ADP | 330 | 498 | 1.5 | 3.10 | 2.50 / 4 |
| T315 | 2GZX | TatD deoxyribonuclease MW0446 from *Staphylococcus Aureus* | 265 | 493 | 0.9 | 2.21 | 1.53 / 5 |
| T317 | 2HCM | Phosphatase | 164 | 508 | 1.1 | 2.83 | 2.14 |
| T318 | 2HB6 | Caenorhabditis elegans leucine aminopeptidase | 491 | 521 | 1.5 | 1.83 | 1.45 / 4 |
| T319 | 2J6A | Zinc finger protein | 141 | 532 | 1.7 | 2.01 | 1.17 / 5 |
| T321 | 2H1Q | Hypothetical protein (ZP_00559375.1) from *Desulfitobacterium Halfniense* | 270 | 505 | 2.4 | 2.64 | 1.89 |
| T322 | 2HBO | Thioesterase superfamily protein | 158 | 512 | 1.7 | 2.78 | 2.05 |
| T324 | 2HDO | Phosphoglycolate phosphatase | 209 | 496 | 1.8 | 1.95 | 1.52 |
| T325 | 2I5I | Putative cellobiose-phosphate cleavage protein | 263 | 494 | 2.2 | 2.85 | 1.44 / 7 |
| T326 | 2H2W | Homoserine O-succinyltransferase | 312 | 476 | 1.0 | 4.01 | 3.88 |
| T329 | 2HI0 | Phosphoglycolate phosphatase | 240 | 523 | 1.7 | 1.87 / 2 | 1.16 / 6 |
| T330 | 2HCF | Hydrolase haloacid dehalogenase-like | 234 | 515 | 2.1 | 2.33 | 1.92 |
| T332 | 2HA8 | Methyltransferase domain of human TAR (HIV-1) RNA binding protein 1 | 184 | 541 | 1.3 | 0.66 / 4 | 0.55 / 6 |
| T334 | 2OAM | Apo RebH from *Lechevalieria Aerocolonigenes* | 550 | 492 | 1.0 | 3.27 | 2.80 |
| T338 | 2IVX | Human Cyclin T2 | 257 | 577 | 1.8 | 1.45 | 0.96 / 3 |
| T339 | 2HDY | Human selenocysteine lyase | 440 | 527 | 1.3 | 1.92 | 1.36 / 4 |
| T340 | 2HE4 | PDZ domain of human NHERF-2 | 90 | 541 | 0.8 | 0.87 / 3 | 0.27 / 6 |
| T341 | 2HO4 | Haloacid dehalogenase-like hydrilase domain | 259 | 508 | 1.7 | 1.79 / 3 | 1.05 / 8 |
| T342 | 2I5T | Hypothetical protein LOC79017 from *Homo Sapiens* | 188 | 518 | 1.7 | 2.39 | 2.05 |
| T345 | 2HE3 | Human glutathionine peroxidase 2 (GPX2) | 208 | 483 | 0.7 | 2.26 | 1.85 |
| T346 | 2HE9 | Peptidylprolyl isomerase domain | 192 | 519 | 0.4 | 0.81 / 4 | 0.57 / 8 |
| T348 | 2HF1 | Tetraacyldisaccharide-1-P 4-kinase | 68 | 556 | 1.4 | 1.59 | 1.22 |
| T354 | 2ID1 | Hypothetical protein CV0518 from *Chromobacterium Violaceum* | 130 | 552 | 2.1 | 2.89 | 2.59 |
| T356 | 2IDB | 3-octaprenyl-4-hydroxybenzoate decarboxylase | 505 | 554 | 2.3 | 2.37 | 2.16 |
| T359 | 2IWN | PDZ domain of MPDZ | 97 | 548 | 1.4 | 0.45 / 5 | 0.15 / 7 |
| T360 | 2HXJ | Hypothetical protein NMB1681 from *Neisseria Meningitidis* | 144 | 502 | 1.7 | 2.91 | 2.12 |
| T362 | 2HX5 | Putative thioesterase from *Prochlorococcus Marinus* | 152 | 544 | 1.5 | 1.76 / 2 | 1.30 / 5 |
| T363 | 2HJ1 | 3D domain-swapped dimer of hypothetical protein from *Haemophilus Influenzae* | 97 | 565 | 1.3 | 3.88 | 3.52 |
| T364 | 2HLJ | Hypothetical protein (NP_742468.1) from *Pseudomonas Putida* | 157 | 538 | 1.5 | 0.67 / 3 | 0.51 / 6 |
| T365 | 2IIU | PhoU-like phosphate regulatory protein | 227 | 526 | 2.1 | 3.12 | 2.73 |
| T366 | 2IWO | 12th PDZ Domain of MPDZ | 120 | 558 | 0.9 | 1.61 / 2 | 0.98 / 5 |
| T367 | 2HSB | Hypothetical protein (NP_069135.1) from Archaeoglobus Fulgidus | 126 | 534 | 1.1 | 3.93 | 3.38 |
| T369 | 2HKV | conserved hypothetical protein of the DinB family | 149 | 533 | 2.1 | 3.33 | 2.84 |
| T370 | 2IAB | Protein with FMN-binding split barrel fold | 155 | 514 | 1.9 | 2.47 | 1.91 |
| T371 | 2HX1 | Possible sugar phosphatase, HAD superfamily | 284 | 516 | 2.2 | 2.27 | 1.53 / 6 |
| T372 | 2HQY | Hypothetical protein from *Bacteroides Thetaiotaomicron* | 305 | 539 | 2.1 | 0.93 / 10 | 0.70 / 15 |
| T374 | 2I6C | Putative acetyltransferase (GNAT family) | 160 | 552 | 1.9 | 1.71 / 2 | 1.14 / 5 |
| T375 | 2HLZ | Human ketohexokinase | 312 | 549 | 1.5 | 0.98 / 3 | 0.56 / 7 |
| T376 | 2HMC | Dihydrodipicolinate synthase DapA | 344 | 534 | 1.8 | 2.67 | 1.00 / 7 |
| T378 | 2I6D | Putative RNA methyltransferase of the TrmH family | 257 | 521 | 1.6 | 2.71 | 2.18 |
| T380 | 2HQ7 | General stress protein 26(GS26) of *B.Subtilis* | 146 | 540 | 1.8 | 3.98 | 2.62 |
| T381 | 2IA2 | Putative transcriptional regulator RHA06195 | 265 | 537 | 0.8 | 3.07 | 2.19 |
| T382 | 2I9C | Hypothetical protein RPA1889 from *Rhodopseudomonas Palustris* | 123 | 563 | 1.2 | 2.88 | 1.74 |
| T383 | 2HNG | Hypothetical protein SP_1558 from *Streptococcus Pneumoniae* | 127 | 608 | 1.9 | 2.27 | 1.26 / 4 |
| T384 | 2HO3 | Oxidoreductase, Gfo/Idh/MocA family | 325 | 537 | 1.8 | 2.11 | 1.49 |
| T385 | 2IB0 | Conserved hypothetical protein, rv2844, from *Mycobacterium Tuberculosis* | 170 | 526 | 1.5 | 2.66 | 1.90 |

Coordinates taken from http://predictioncenter.org/casp7/.

^a^Number of predicted structure submitted in the CASP7 experiment. Column heads are explained in Table S2 footnote.
